# Malignant Peripheral Nerve Sheath Tumor of the Rectum Characterized by Focal S100 Protein Expression

**DOI:** 10.7759/cureus.87235

**Published:** 2025-07-03

**Authors:** Antonio de Jesús González Luna, Cristina Vanessa Cuevas Calla, Christian Daniel Castrejón Cardona, José Alejandro Sánchez García, Ricardo Hernández Ibarra

**Affiliations:** 1 Department of General Surgery, Regional Hospital “Dr. Valentin Gomez Farias”, Institute of Security and Social Services for the State Workers (ISSSTE), Zapopan, MEX; 2 Department of General Surgery, Autonomous University of Guadalajara, Zapopan, MEX; 3 Department of Coloproctology, Regional Hospital “Dr. Valentin Gomez Farias”, Institute of Security and Social Services for the State Workers (ISSSTE), Zapopan, MEX

**Keywords:** immunohistochemistry, malignant peripheral nerve sheath tumor, mpnst, peripheral nervous system neoplasms, rectal neoplasms, s100 proteins, sarcoma

## Abstract

This case describes a malignant peripheral nerve sheath tumor (MPNST) arising in the ischiorectal fossa, an anatomical location that is infrequently reported in the scientific literature. The report provides new anatomical and clinical insights into a rare and aggressive neoplasm that is frequently misdiagnosed due to nonspecific symptoms and overlapping histopathological features with other rectal or perianal tumors.

A 52-year-old woman with no significant medical or oncologic history presented with mild rectal bleeding and a painful perianal mass, initially presumed to be thrombosed hemorrhoids. Physical examination revealed a tender, erythematous anal region with prolapsed tissue and persistent bleeding. She underwent a Ferguson hemorrhoidectomy and examination under anesthesia as the initial surgical approach. Intraoperatively, a friable exophytic lesion was identified 4 cm from the anal verge, occupying approximately 70% of the rectal lumen. Biopsies initially suggested a well-differentiated squamous carcinoma with sarcomatoid features.

Further imaging and histopathological evaluation led to an abdominoperineal resection with hysterectomy due to vaginal invasion. Final pathology revealed a high-grade spindle cell neoplasm with lymphovascular and perineural invasion. Immunohistochemistry showed focal S100 positivity (in an irregular pattern) and negativity for AE1/AE3, HMB45, smooth muscle actin (SMA), and CD117. Although SOX10 immunostaining and molecular testing were not performed due to institutional limitations and lack of access to advanced diagnostic resources, the diagnosis of MPNST was supported by compatible histological features and a broad immunohistochemical panel, including markers such as BCL2, CD34, CDX2, P63, CK7, and CK20, and a pan melanoma panel. This combination of findings effectively ruled out major differential diagnoses such as sarcomatoid carcinoma, melanoma, and gastrointestinal stromal tumors (GIST), supporting the final diagnosis of MPNST.

The patient recovered uneventfully from surgery. This case illustrates the diagnostic complexity of MPNST in atypical anorectal locations and emphasizes the need for thorough histopathological and immunohistochemical assessment. Early recognition and complete surgical excision are crucial for improving prognosis in such rare presentations. The limitations related to unavailable molecular testing were acknowledged in the discussion section.

## Introduction

Malignant peripheral nerve sheath tumor (MPNST) is an aggressive mesenchymal neoplasm, accounting for approximately 5-10% of all soft tissue sarcomas and around 0.001% of all malignancies [1]. Despite its rarity, MPNST is clinically significant due to its infiltrative biological behavior, high risk of local recurrence, and limited response to conventional adjuvant therapies. The most common anatomical sites include the retroperitoneum (32.4%), spinal cord (24.4%), and cranial nerves (15.0%) [2]. In contrast, rectoanal involvement is exceedingly rare, with pelvic presentations being exceptionally uncommon; to date, only a few cases arising in the ischiorectal fossa have been reported [3].

Accurate diagnosis of MPNST requires detailed histopathological assessment, typically revealing spindle cell morphology, a high mitotic index, and areas of necrosis. Immunohistochemical confirmation is essential [4]. While S100 protein expression is a helpful marker, it is often focal or patchy and present in only up to 50% of cases [5]. These diagnostic challenges often lead to delayed recognition, particularly when patients present with nonspecific symptoms such as a deep-seated, painless soft tissue mass that gradually enlarges, frequently mistaken for benign conditions like hemorrhoidal disease [6].

In this context, molecular testing has emerged as a valuable adjunct in the diagnosis and prognostic stratification of MPNST, especially in rare or histologically ambiguous presentations. As described by Lee et al. [7], recurrent alterations in genes of the PRC2 complex (e.g., EED and SUZ12) significantly contribute to the characterization of these tumors.

The primary therapeutic approach for MPNSTs is wide surgical resection with negative margins. However, even after radical excision, the risk of locoregional recurrence and distant metastasis remains high, especially in high-grade tumors or those exhibiting perineural or lymphovascular invasion. These tumors are known to follow an aggressive clinical course, with reported local recurrence rates ranging from 40% to 65% and early metastasis occurring in up to 68% of cases [5]. Adjuvant chemotherapy and radiotherapy have not consistently shown survival benefits, though they may be considered in advanced or unresectable cases [8]. Recently, increasing attention has been directed toward molecular biomarkers and potential therapeutic targets, including alterations in the RAS/MAPK and mTOR pathways, although these remain under clinical investigation [8].

Herein, we present a case of a 52-year-old woman with no oncologic history who initially presented with symptoms suggestive of hemorrhoidal disease. Subsequent surgical and histopathological evaluation revealed a rectoanal MPNST characterized by high-grade spindle cell morphology and focal S100 positivity. This case underscores the importance of including this rare neoplasm in the differential diagnosis of aggressive rectoanal tumors.

## Case presentation

We present a case of a 52-year-old woman with no relevant personal or family history of oncologic disease, chronic-degenerative conditions, or neurofibromatosis type 1 (NF1). Her surgical history included two cesarean deliveries (in 2003 and 2008). She presented to the emergency department with mild rectal bleeding during defecation, initially attributed to hemorrhoidal disease. She also reported a soft, painful perianal mass that was initially transient but later became persistent, associated with chronic constipation and progressive abdominal pain. Over time, she developed protrusion of a painful mass accompanied by continuous rectal bleeding, which necessitated medical evaluation.

On physical examination, the anal region was erythematous and tender, with a thrombosed prolapsed hemorrhoid and rectal bleeding not attributable to the presumed diagnosis. Digital rectal examination was deferred because of the patient’s severe discomfort. Analgesia was initiated, and a proctology consultation was requested. The patient was hemodynamically stable, with no signs of peritoneal irritation.

Laboratory studies revealed hemoglobin at 12 g/dL (12.6-16.6), leukocytes at 13.98 ×10³/µL (4.50-10.50), neutrophils at 11.9 ×10³/µL (2.50-7.00), platelets at 605 ×10³/µL (150.0-420.0), and normal renal and hepatic function.

**Figure 1:**
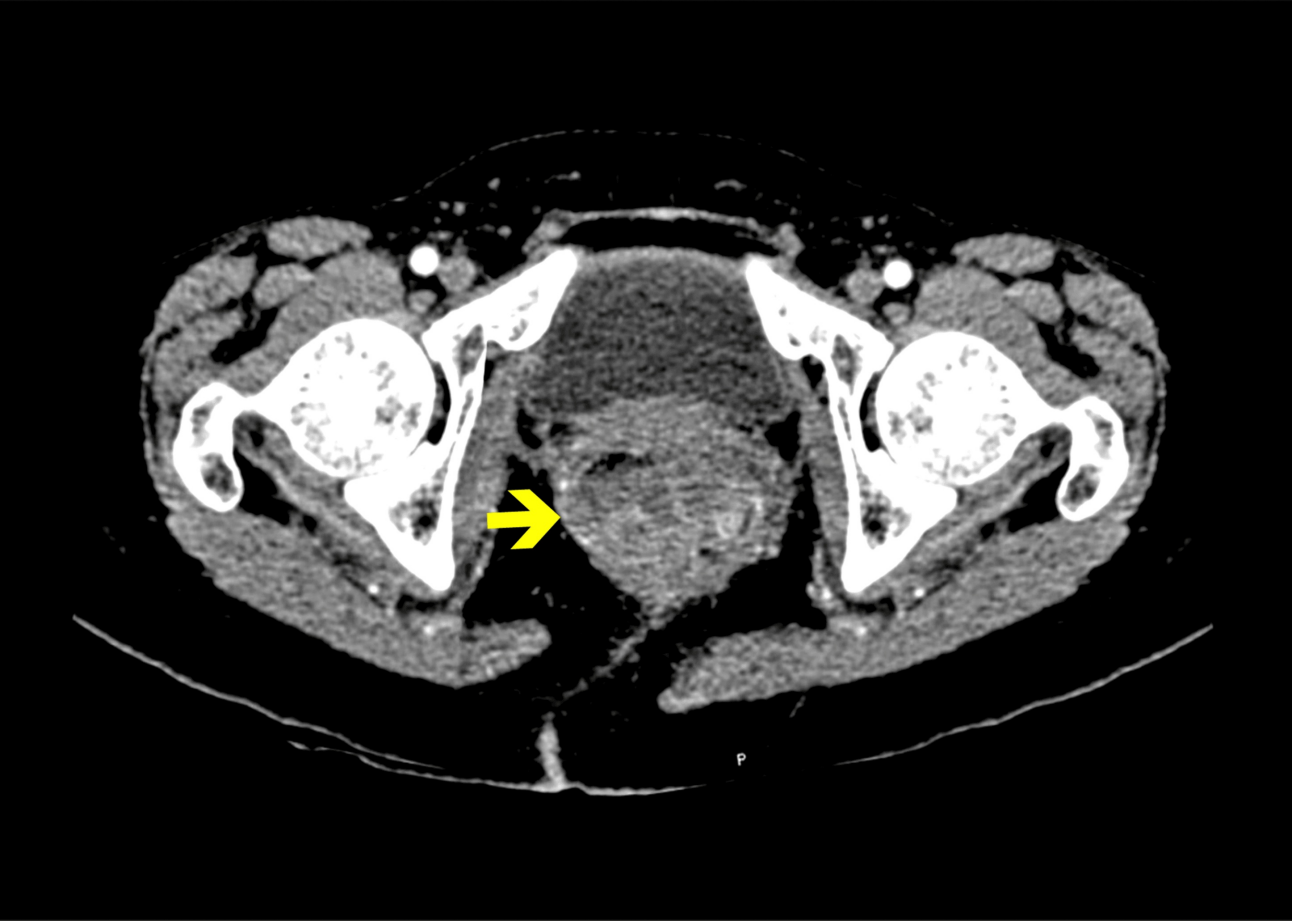
Axial CT image (contrast-enhanced). Axial contrast-enhanced computed tomography (CT) of the pelvis showing a soft tissue mass occupying the rectal lumen, with irregular borders and apparent infiltration into the mesorectal fat. The lesion appears hypodense compared to the surrounding muscle tissue. There is no clear evidence of pelvic bone erosion. Bilateral femoral heads and acetabula are intact.

**Figure 2:**
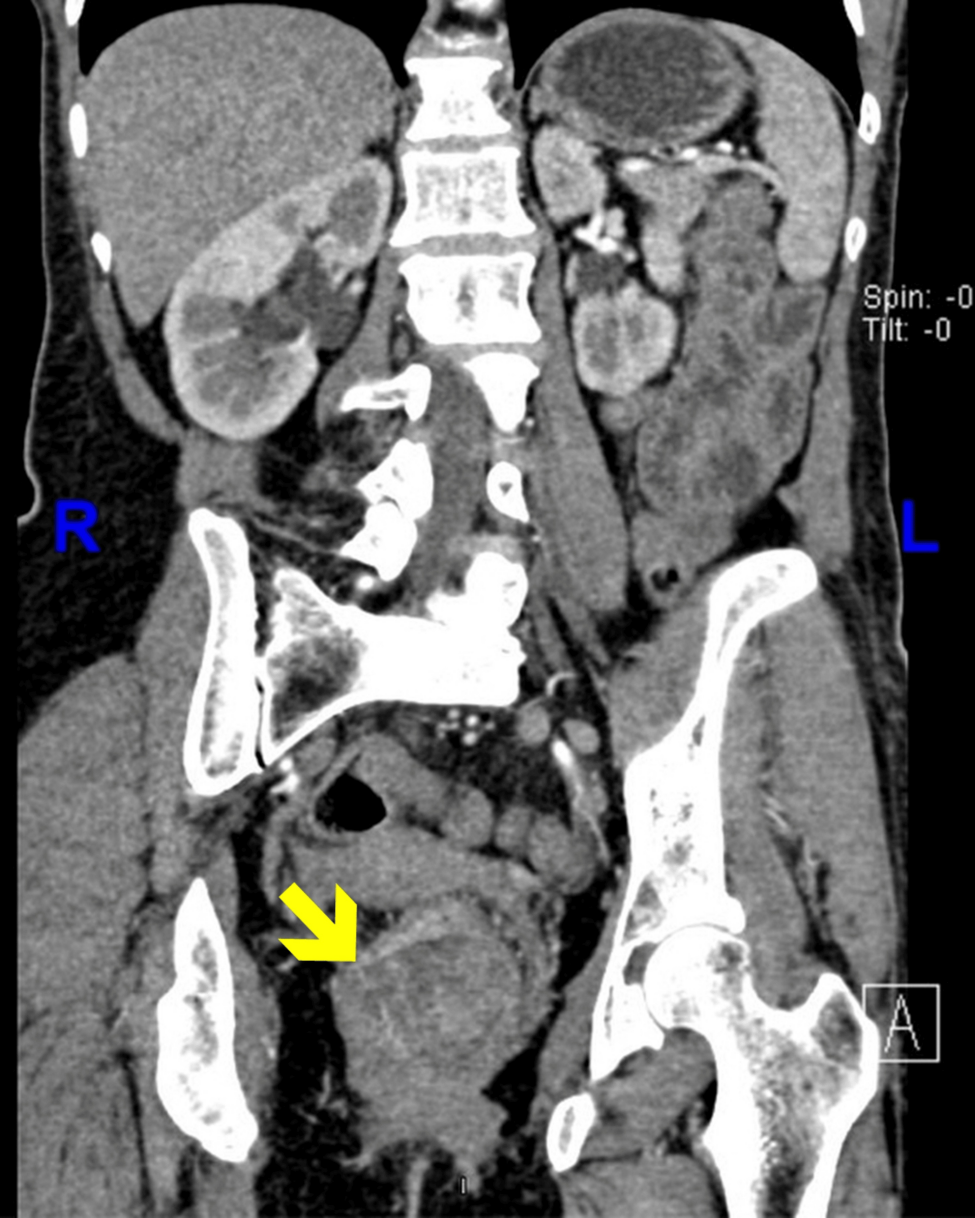
Coronal CT image (contrast-enhanced). Coronal contrast-enhanced CT scan of the abdomen and pelvis showing an irregular soft tissue mass within the lower pelvis, below the peritoneal reflection. The lesion displaces adjacent bowel loops and appears to distort the rectosigmoid contour. No radiographic evidence of hepatic metastases or retroperitoneal lymphadenopathy is observed.

**Figure 3:**
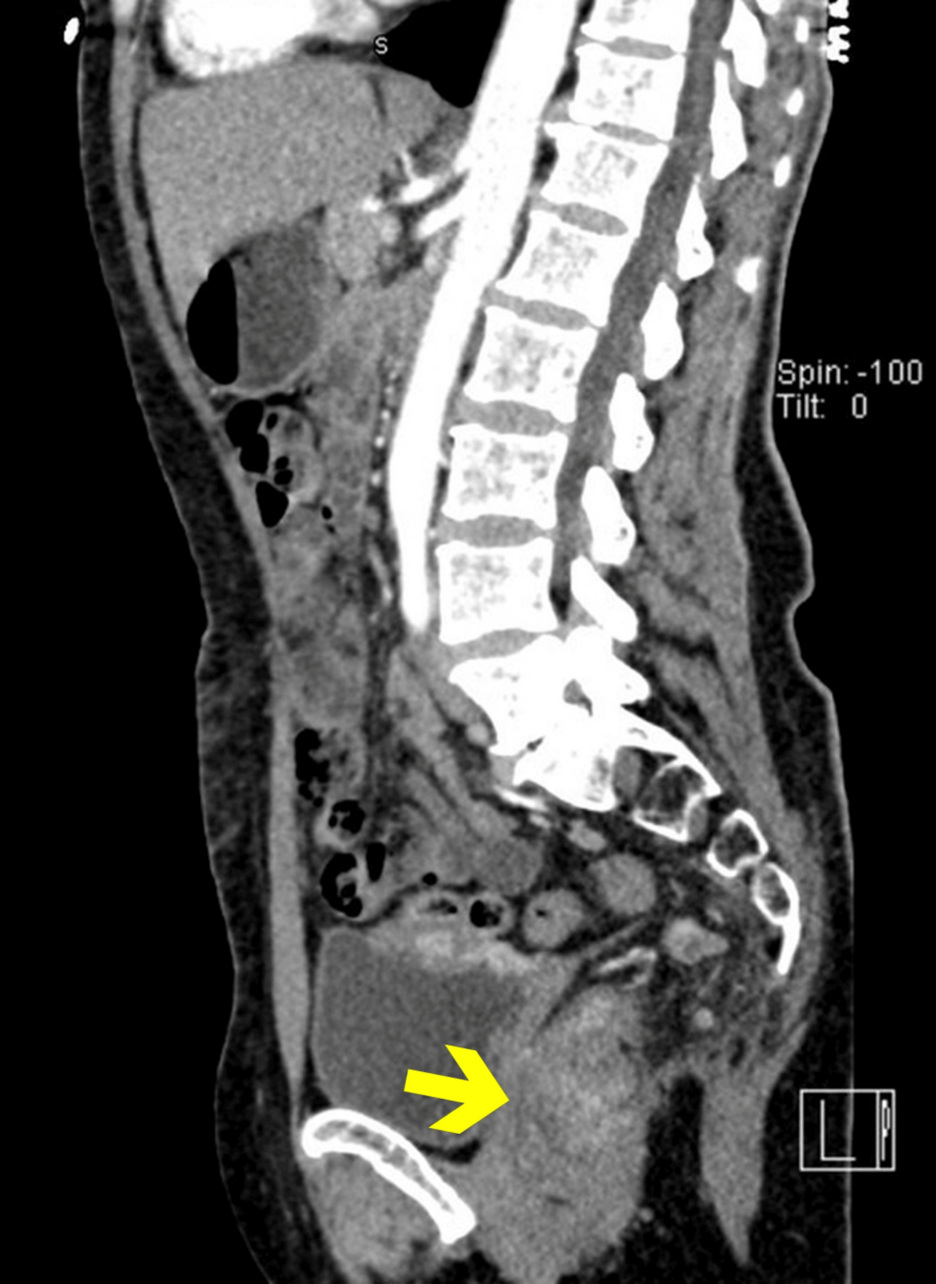
Sagittal CT image (contrast-enhanced). Sagittal CT reconstruction demonstrates an irregular mass centered in the rectum, with involvement of the rectal wall and partial obliteration of the fat planes with surrounding structures. The mass lies below the peritoneal reflection, without signs of bladder invasion or presacral bone destruction.

She was taken to the operating room for Ferguson hemorrhoidectomy and examination under anesthesia. Intraoperatively, a friable, hemorrhagic, exophytic lesion was identified 4 cm from the anal verge, occupying approximately 70% of the rectal circumference and lumen. Biopsies were taken, and she was discharged with outpatient follow-up and pending histopathology and CT imaging (Figures 1-3).

The initial biopsy, performed externally and not available for institutional review, reported a well-differentiated squamous cell carcinoma with sarcomatoid features. Due to this preliminary diagnosis and the need for prompt surgical planning, magnetic resonance imaging (MRI) was not performed preoperatively. Additionally, limited institutional access to advanced imaging modalities in non-confirmed oncologic settings contributed to this decision. An abdominoperineal resection was scheduled accordingly, and the final pathology later revealed an MPNST.

Intraoperatively, a rectosigmoid mass with invasion of the vaginal dome was confirmed, requiring total hysterectomy with partial vaginal resection. An en bloc abdominoperineal resection was successfully performed.

Histological examination of the resected specimen revealed a poorly differentiated malignant neoplasm with both epithelioid and sarcomatoid morphology, associated with lymphovascular and perineural invasion, as well as significant nodal involvement (14 of 17 lymph nodes positive). These findings were initially interpreted as an epithelial neoplasm with sarcomatoid differentiation.

All surgical margins were free of tumor. Although the neoplasm protruded through a mesorectal incision, there was no direct invasion of the circumferential resection margins. The anterior circumferential margin was 0.5 cm from the tumor, while the posterior margin was 2 cm away.

**Figure 4:**
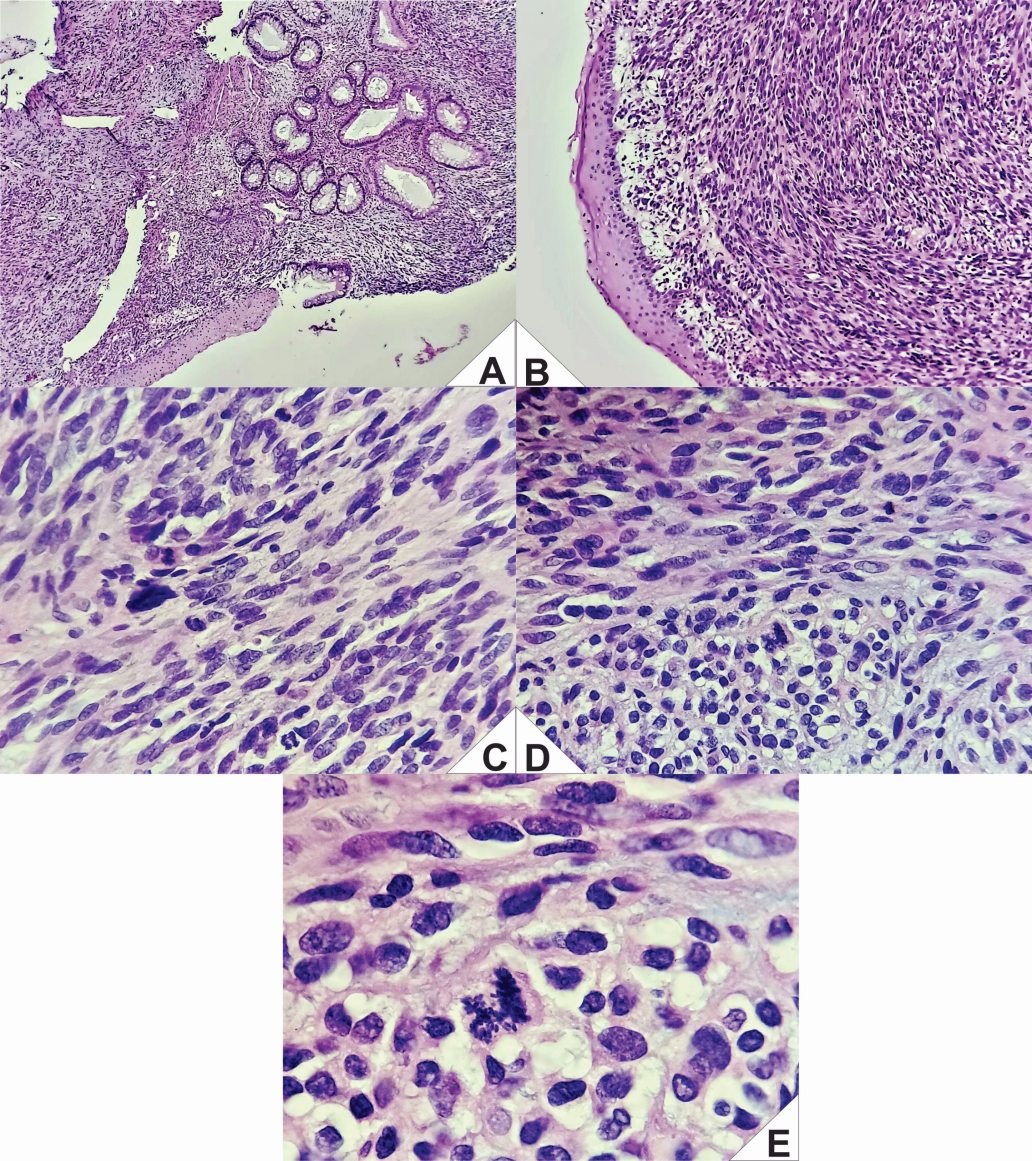
Representative histopathological findings (hematoxylin and eosin, H&E). A: Transition zone of the rectoanal squamocolumnar junction. Mucus-secreting columnar and keratinized squamous epithelium is observed, with loss of mucosal architecture and the presence of atypical cells in the underlying stroma. B: Proliferation of spindle cells arranged in parallel bundles within a dense stromal background. C: Spindle cells exhibiting mild to moderate pleomorphism, with elongated and hyperchromatic nuclei. D: Higher magnification showing nuclear pleomorphism and disorganized cellular arrangement. E: High-power field demonstrating markedly pleomorphic cells, with bizarre, hyperchromatic nuclei and frequent atypical mitotic figures.

Subsequent pathological reevaluation identified a proliferation of spindle cells with a storiform pattern, marked cellular pleomorphism, and high mitotic activity (31 mitoses per 10 high-power fields) (Figure 4). This morphology was suggestive of a high-grade undifferentiated sarcoma.

**Figure 5:**
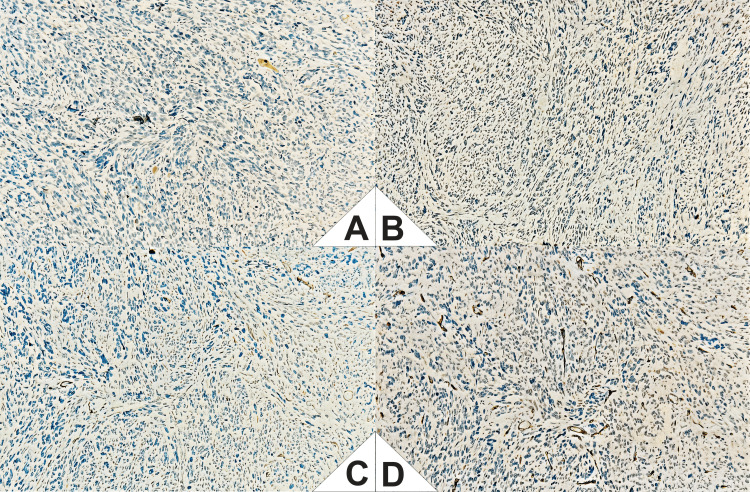
Immunohistochemical panel (IHC). A (S100): Demonstrated focal cytoplasmic positivity in discrete clusters of tumor cells, arranged in a “patchy” distribution throughout the neoplastic tissue. Staining intensity was moderate, with preservation of nuclear morphology. The positivity was not diffuse and was restricted to isolated areas within the observed field. B (CD117): Showed negative staining in the tumor cell population. No cytoplasmic or membranous enhancement was observed in neoplastic cells. Background structural details were preserved, with no evidence of nonspecific staining. Vascular and other internal structures also lacked appreciable immunoreactivity. C (SMA): Showed negative staining in tumor cells. However, focal cytoplasmic positivity was identified in intratumoral vascular structures, specifically in the walls of small-caliber blood vessels. The staining was of moderate intensity and was exclusively localized to the smooth muscle component of the vessel walls. D (CD34): Demonstrated positive expression in stromal blood vessels, evidenced by cytoplasmic and membranous staining in endothelial cells outlining the vascular architecture. No positivity was observed in neoplastic cells. The staining was confined to the vasculature, with no background immunoreactivity or nonspecific staining.

The initial immunohistochemical panel was negative for S100, CD117, CD34, and smooth muscle actin (SMA), which precluded definitive classification at that time (Figure 5). However, it was noted that S100 expression can be focal or "patchy," and thus a neural origin could not be entirely ruled out.

Expanded immunohistochemical analysis of the resected specimen confirmed the following: S100: focal cytoplasmic positivity with moderate intensity ("patchy" pattern). AE1/AE3, HMB45, BCL2, and pan-melanoma: negative, excluding epithelial, melanocytic, and lymphoid origin.

Taken together, the spindle cell morphology, high mitotic index, lack of expression of other lineage markers, and focal S100 positivity allowed for a definitive diagnosis of MPNST.

The patchy S100 expression was a key finding for the final diagnosis, underscoring the importance of considering this entity in the differential diagnosis of aggressive spindle cell rectoanal neoplasms with atypical morphology.

Although molecular testing (e.g., loss of H3K27me3) has emerged as a valuable tool in the diagnosis and prognostic stratification of MPNST, no molecular studies were performed in this case due to limited access to advanced diagnostic resources at our institution.

The immediate postoperative course was uneventful, and the patient was discharged on postoperative day seven with scheduled outpatient follow-up. At eight months, imaging revealed local tumor recurrence involving the perianal and intergluteal regions, deemed unresectable due to its infiltrative nature. Although no distant metastases were initially detected, the aggressive biological behavior of the MPNST prompted the initiation of palliative external beam radiotherapy.

A total dose of 80 Gy was administered in 40 fractions using intensity-modulated radiation therapy (IMRT), with good tolerance and no major complications. However, subsequent follow-up revealed systemic disease progression, with multiple hepatic, pulmonary, and pelvic metastases. Given the poor prognosis and the patient's functional status, palliative systemic therapy with pazopanib was initiated, supported by available evidence in advanced soft tissue sarcomas.

Unfortunately, the patient died approximately one year after surgery due to widespread metastatic disease and multiorgan failure.

## Discussion

This case, which documents an MPNST located in the ischiorectal region, expands the known anatomical spectrum of this tumor type. While most MPNSTs originate from major peripheral nerves, typically in the extremities, plexuses, and trunk, their occurrence in deep anatomical spaces such as the ischiorectal fossa is extremely rare [3,6]. Although cases involving the pelvis or other visceral sites have been reported, no prior publications have provided a detailed description of MPNSTs originating specifically in the ischiorectal area. A recently reported case involving a 61-year-old woman, in which complete resection was achieved via a transperineal approach, supports the notion that, despite its rarity, localized surgical management can be successful [3].

From a diagnostic standpoint, this case aligns with previous literature highlighting the frequent difficulty in achieving a definitive preoperative diagnosis. This is largely due to nonspecific clinical presentations, such as soft perianal masses or mild rectal bleeding, that are often mistaken for benign conditions like hemorrhoids or lipomas [3]. In our case, the initial diagnosis of squamous cell carcinoma with sarcomatoid features emphasized the need for thorough pathological reevaluation, as reflected in other reports of non-epithelial rectal neoplasms ultimately diagnosed as MPNSTs [9].

From a diagnostic perspective, this case highlights the critical importance of early multidisciplinary evaluation in soft tissue sarcomas with atypical anorectal presentations. Timely collaboration among colorectal surgeons, oncologists, radiologists, and pathologists is essential to ensure diagnostic precision and guide therapeutic decisions. Although preoperative MRI was not performed due to institutional limitations, which may have impacted staging and surgical planning, the final diagnosis was successfully established through comprehensive histopathological and immunohistochemical analysis. This case exemplifies the inherent challenges in diagnosing rare anorectal sarcomas in resource-limited settings and underscores the need for broader diagnostic access in such scenarios.

Immunohistochemically, the focal S100 expression and negativity for epithelial, melanocytic, and muscular markers observed in this case are consistent with recent series [10]. Although H3K27me3 loss, recognized as a highly valuable diagnostic and prognostic biomarker in MPNST, was not assessed in this case due to technical limitations at our institution, its routine use is recommended, given reported sensitivities ranging from 30% to 80%, depending on tumor grade [10].

Radical treatment with wide surgical excision and negative margins reflects the current best evidence; even in deep-seated locations, aggressive surgery remains essential and may be complemented by radiotherapy [4,8]. In our patient, the decision to perform an en bloc abdominoperineal resection along with partial vaginal resection was driven by the presence of local tumor invasion. This strategy ensured negative margins and minimized the risk of local recurrence. Literature supports that combined approaches may enhance local control, although the effect on long-term survival remains uncertain [2,4,8].

The novel contribution of this case lies in the documented occurrence of an MPNST strictly confined to the ischiorectal fossa, with favorable clinical outcomes achieved through a multidisciplinary approach that included diagnostic accuracy through targeted morphological and immunohistochemical analysis, specialized surgical planning, enabling conservative en bloc resection, and an integrated therapeutic strategy, including terminal colostomy and potential adjuvant radiotherapy.

These elements underscore the importance of including MPNST in the differential diagnosis of aggressive spindle cell tumors encountered in emergency or outpatient settings and highlight the need to consider biopsy in atypical perianal masses [3], as well as early referral to centers specializing in soft tissue tumors.

The main limitations of this case include the unavailability of advanced molecular testing (e.g., NF1, BRAF, and H3K27me3) and the lack of long-term follow-up, both factors identified in the literature as relevant for prognostication and the selection of emerging therapies [10]. Future reports should aim to incorporate genomic analyses and proliferation markers (e.g., Ki-67 and p53), which have been associated with poorer outcomes [11], and to document clinical responses to targeted therapies currently under investigation.

## Conclusions

MPNSTs are rare and aggressive neoplasms that may initially present with nonspecific symptoms, especially when arising in anatomically uncommon locations such as the ischiorectal fossa. This case illustrates how misdiagnosis as a benign anorectal condition can lead to delays in appropriate treatment and highlights the importance of maintaining a high index of suspicion in atypical presentations.

Timely biopsy, comprehensive histopathological evaluation, and immunohistochemical analysis, including recognition of focal or patchy S100 expression, are essential for accurate diagnosis. Optimal outcomes depend on prompt surgical intervention with wide excision margins and the involvement of a multidisciplinary team. This report adds valuable insight into the diagnostic challenges and therapeutic considerations of MPNSTs arising in complex pelvic regions and underscores the need for increased clinical awareness of this rare but serious malignancy.
